# Comparison of estimates and time series stability of Korea Community Health Survey and Korea National Health and Nutrition Examination Survey

**DOI:** 10.4178/epih.e2019012

**Published:** 2019-04-07

**Authors:** Ji Son Ki, Ho Kim

**Affiliations:** 1 College of Nursing, Seoul National University, Seoul, Korea; 2 Graduate School of Public Health, Seoul National University, Seoul, Korea

**Keywords:** Korea Community Health Survey, Korea National Health and Nutrition Examination Survey, Estimates, Comparison, Time series stability

## Abstract

**OBJECTIVES:**

In South Korea, there are two nationwide health surveys conducted by the Korea Centers for Disease Control and Prevention: the Korea Community Health Survey (KCHS) and Korea National Health and Nutrition Examination Survey (KNHANES). The two surveys are directly comparable, as they have the same target population with some common items, and because both surveys are used in various analyses, identifying the similarities and disparities between the two surveys would promote their appropriate use. Therefore, this study aimed to compare the estimates of six variables in KCHS and eight variables in KNHANES over a six-year period and compare time series stability of region-specific and sex- and age-specific subgroup estimates.

**METHODS:**

Data from adults aged 19 years or older in the 2010-2015 KCHS and KNHANES were examined to analyze the differences of estimates and 95% confidence interval for self-rated health, current smoking rate, monthly drinking rate, hypertension diagnosis rate, diabetes diagnosis rate, obesity prevalence, hypertension prevalence, and diabetes prevalence. The variables were then clustered into subgroups by city as well as sex and age to assess the time series stability of the estimates based on mean square error.

**RESULTS:**

With the exception of self-rated health, the estimates taken based on questionnaires, namely current smoking rate, monthly drinking rate, hypertension diagnosis rate, and diabetes diagnosis rate, only differed by less than 1.0%p for both KCHS and KNHANES. However, for KNHANES, estimates taken from physical examination data, namely obesity prevalence, hypertension prevalence, and diabetes prevalence, differed by 1.9-8.4%p, which was greater than the gap in the estimates taken from questionnaires. KCHS had a greater time series stability for subgroup estimates than KNHANES.

**CONCLUSIONS:**

When using the data from KCHS and KNHANES, the data should be selected and used based on the purpose of analysis and policy and in consideration of the various differences between the two data.

## INTRODUCTION

Public health questionnaire surveys are the foundation of modern epidemiology and play an important role in devising policies and programs for the promotion of public health and prevention of diseases [1]. These surveys are conducted periodically, and accurate health survey data are highly useful for understanding the scope and trends of health problems [2]. In particular, researchers and policymakers are able to understand and predict current and future health problems and facilitate effective use of limited resources based on these data [3].

The two classic public health surveys conducted in South Korea (hereafter Korea) are Korea Community Health Survey (KCHS) and Korea National Health and Nutrition Examination Survey (KNHANES) controlled by the Korea Centers for Disease Control and Prevention (KCDC). KCHS presents health statistics in units of city, *gun*, and *gu* required for establishing community healthcare plans, thereby enabling interregional comparisons and serving as indices of community health projects [4]. KNHANES computes national statistics for people’s health, health-related awareness and behavior, and food and nutrition intake and is used for goal-setting and assessment of the Health Plan. Further, it provides national statistical data requested by the World Health Organization (WHO) and the Organization for Economic Cooperation and Development, such as smoking, drinking, physical activity, and obesity data [5]. The target population for both surveys is Korean citizens, and they both include the entire region of Korea. Furthermore, they share many survey items although the two surveys serve different purposes.

In foreign countries, the estimates of surveys with different purposes but having duplicate items are continually compared to assess the validity of the surveys. The most accurate method to validate survey estimates is to examine the entire population, but this is practically impossible; therefore, these estimates can be compared with those of other surveys [6]. In the USA, studies have compared self-rated health estimates among four national health surveys, namely Behavioral Risk Factor Surveillance System (BRFSS), Current Population Survey, National Health and Nutrition Examination Survey (NHNES), and National Health Interview Survey [7], binge drinking rate estimates between BRFSS and National Survey on Drug Use and Health [8], and obesity estimates between BRFSS and NHNES [9].

However, there is a lack of studies comparing the estimates between different national surveys in Korea. This is because KCHS and KNHANES are virtually the only two national health surveys and because of their different fundamental purposes, assessing the validity of one survey with reference to the other survey is controversial. However, both surveys are actively utilized for policymaking and research and for diverse analyses in deviation from their purposes [10-13]. Identifying the similarities and disparities between the estimates of two surveys would promote appropriate use of the data.

Policymakers and researchers would be able to understand current health problems more accurately if they select the relevant data or appropriately utilize both data based on data features and the purpose of their analysis, as opposed to their convenience.

Therefore, this study aimed to compare estimates between KCHS and KNHANES, two surveys that serve as the foundation for computing important national statistics. To this end, we compared the estimates for six questionnaire variables in KCHS and eight questionnaire and physical examination variables in KNHANES over a six-year period and analyzed the time series stability of estimates of city-specific and sex- and age-specific subgroups.

## MATERIALS AND METHODS

### Study data

Data from KCHS and KNHANES between 2010 and 2015 were used. Both surveys were conducted by the KCDC. KCHS were collected via an interview with all adult members (aged≥ 19 years) of the sample households. Nine hundred people per city, *gun*, and *gu* are surveyed, for a total of 220,000 people every year. Sampling is performed to ensure proportional sampling probability in consideration of household sizes based on the number of households by home type within *tong*, *ban*, and *ri*, and secondary sample households are selected via systematic sampling [4]. KNHANES is conducted on the members (≥ 1 year) of sample households, with about 10,000 people surveyed every year. Similar to KCHS, sampling is performed with a complex sample design with city/province, *dong/eup/myeon*, and home type as the stratification variables. A total of 3,840 households in 192 districts are chosen every year, and the members are classified as children (aged 1-11 years), adolescents (aged 12-18 years), and adults (aged ≥ 19 years). Age-specific items are used for each group, and health questionnaire, physical examination, and nutritional survey are administered for all participants [5].

In this study, participants were limited to adults aged 19 years or older, and the number of participants is shown in Table 1. Although the number of subjects to be surveyed shows a difference of about 40 times every year between KCHS and KNHANES, the weighted number of subjects is similar between the two surveys after applying weighted values to represent the target population —the Korean population—considering that a complex sample design was used for both surveys. The weighted number of subjects differed by about 2% between 2010 and 2013 and by 1% from 2014 to 2015, an average of 2% difference over six years.

When the weighted subjects are divided into subgroups by city, as well as sex and age, there is an average of 9% and 5% difference with reference to the 2015 current smoking rate. The differences vary across years and variables, and arise from non-responses. In the present study, we considered these differences as a feature of the data and thus analyzed the data as is without age standardization.

### Definition of variables

To minimize bias, we selected the same variables that the question can correspond to the two surveys. From KCHS, the following six variables were analyzed: self-rated health, current smoking rate, monthly drinking rate, hypertension diagnosis rate, diabetes diagnosis rate, and obesity prevalence. From KNHANES, the following eight variables were analyzed: self-rated health, current smoking rate, monthly drinking rate, hypertension diagnosis rate, diabetes diagnosis rate, obesity prevalence, hypertension prevalence, and diabetes prevalence. Obesity prevalence, hypertension prevalence, and diabetes prevalence in KNHANES were analyzed using physical examination data, and the remaining variables were analyzed based on questionnaire data.

Self-rated health was defined as the percentage of participants who perceived their health to be very good or good, and current smoking rate was defined as the percentage of participants who claimed to have smoked at least five packs (100 cigarettes) in their lifetime and currently smoke every day or occasionally. Monthly drinking rate referred to the percentage of participants who have drank at least one shot of drink in their lifetime and currently drink at least once a month. In KCHS, hypertension diagnosis rate and diabetes rate referred to the percentage of participants who had been diagnosed with hypertension and diabetes, respectively, by a physician. In KNHANES, hypertension diagnosis rate and diabetes diagnosis rate referred to the percentage of participants who responded “yes” to the question asking whether they have been diagnosed with hypertension and diabetes, respectively, by a physician.

KCHS measured body mass index (BMI) using self-reported height and weight, while KNHANES measured BMI using height and weight measured during physical examination. Obesity was defined as a BMI of 25 kg/m^2^ or greater based on the WHO AsiaPacific criteria for obesity [14], and obesity rates were compared between the two sets of data. Hypertension prevalence in KNHANES was defined as a systolic blood pressure (BP) of 140 mmHg or higher or diastolic BP of 90 mmHg higher for three repeated BP measurements in a physical examinations or the use of hypertension drugs. The hypertension prevalence was compared with hypertension diagnosis rate in KCHS. Diabetes prevalence in KNHANES was defined as a fasting (≥ 8 hours) blood glucose level of 126 mg/dL or higher measured in a physical examination, diagnosis by a physician, use of hypoglycemic agent, or use of insulin injections, and diabetes prevalence was compared with the diabetes diagnosis rate in KCHS. Because hypertension and diabetes are chronic diseases that can be controlled, as opposed to being cured, we deemed it appropriate to conclude individuals to have the disease if they had ever been diagnosed with it in their lifetime. Also, it would be appropriate to compare this estimate with an objective assessment of the disease based on health examination data.

### Statistical analysis

Because both surveys used a complex sample design, we considered weight, stratification and clustering for the computation of the estimates. When comparing the estimates, the absolute difference, 95% confidence interval (CI) of the difference, and relative difference (ratio of absolute difference to mean KNHANES estimate) were analyzed for the annual estimates for each variable. The absolute difference is the absolute value of the difference between estimates, and relative difference is the proportion of absolute difference in the mean estimate. Variables with low estimates tend to have low absolute differences, so examining relative difference more clearly shows the difference between estimates regardless of the size of the estimates.

Time series stability was compared among 16 city subgroups, excluding the city of Sejong, which was not separately surveyed until the sixth KNHANES (2013-2015), and among 14 sex and age subgroups (19-88 years divided into 10-year units for males and females). A simple linear regression line was computed using six years of estimates by subgroup, and the variability of the estimates were assessed using mean square error (MSE) of the estimates to the line. For example, if there is low variability in the estimates over six years, the MSE would be lower, and this would indicate high time series stability. All analyses were performed using SAS version 9.4 (SAS Institute Inc., Cary, NC, USA) and R version 3.4.4 (https://cran.r-project.org/bin/windows/base/old/3.4.4/).

### Ethical statement

This study was waived for review by the Institutional Review Board (IRB) at Seoul National University (IRB No. E1711/003-002).

## RESULTS

### Comparison of estimates by variable between KCHS and KNHANES

**Figure 1.**
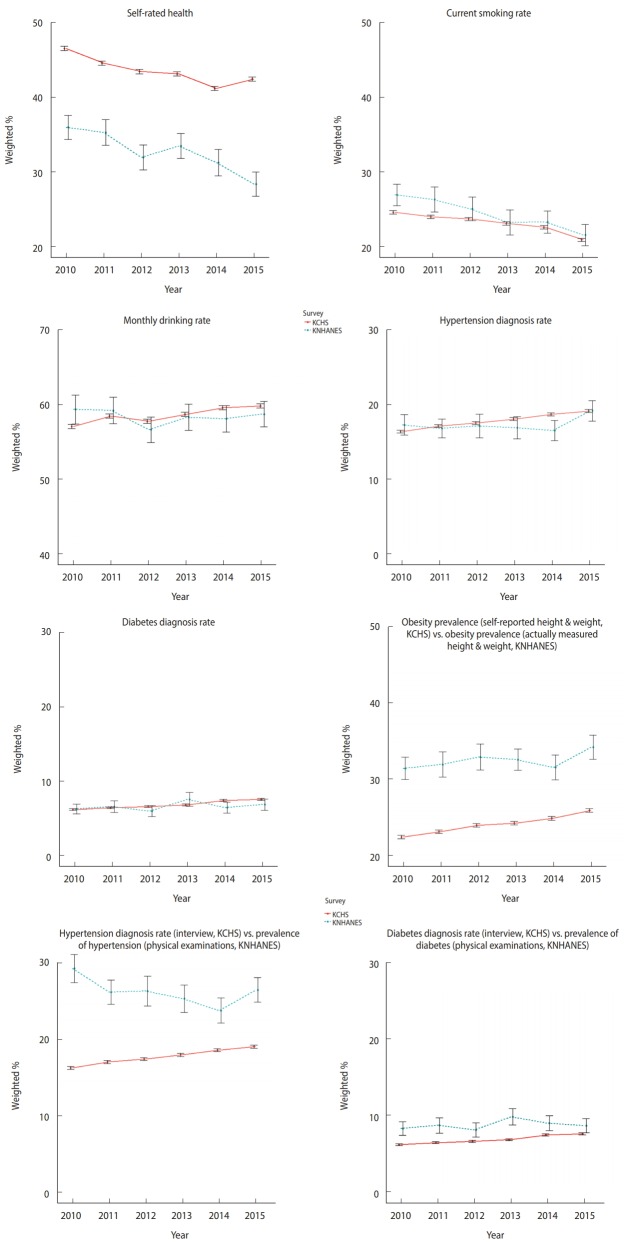
Trends in the variable estimates of Korea Community Health Survey (KCHS) and Korea National Health and Nutrition Examination Survey (KNHANES), 2010-2015.

Figure 1 and Table 2 show the comparison of estimates by variable between both surveys over six years from 2010 to 2015.

The mean absolute difference of self-rated health over six years was 10.8%p, with 10.5%p in 2010, 9.2%p in 2011, 11.4%p in 2012, 9.6%p in 2013, 9.9%p in 2014, and 14.0%p in 2015. The mean relative difference was 33.0%p. The differences were greater than those for other variables.

The mean absolute difference of current smoking rate over six years was 1.2%p, with 2.3%p in 2010, 2.3%p in 2011, 1.3%p in 2012, 0.1%p in 2013, 0.6%p in 2014, and 0.6%p in 2015. The mean relative difference was 4.9%p.

The mean absolute difference of monthly drinking rate over six years was 1.1%p, with 2.2%p in 2010, 0.7%p in 2011, 1.1%p in 2012, 0.4%p in 2013, 1.4%p in 2014, and 1.0%p in 2015. The mean relative difference was 1.9%p.

The mean absolute difference of hypertension diagnosis rate over six years was 0.8%p, with 0.8%p in 2010, 0.3%p in 2011, 0.3%p in 2012, 1.1%p in 2013, 2.1%p in 2014, and 0.0%p in 2015. The mean relative difference was 4.6%p.

The mean absolute difference of diabetes diagnosis rate over six years was 0.6%p, with 0.1%p in 2010, 0.2%p in 2011, 0.6%p in 2012, 0.8%p in 2013, 1.0%p in 2014, and 0.7%p in 2015. The mean relative difference was 9.0%p.

The mean absolute difference between obesity prevalence by self-reported height and weight in KCHS and by actually measured height and weight in KNHANES over six years was 8.3%p, with 8.9%p in 2010, 8.7%p in 2011, 8.8%p in 2012, 8.3%p in 2013, 6.6%p in 2014, and 8.2%p in 2015. The mean relative difference was 25.6%p.

The mean absolute difference between hypertension diagnosis rate by interview in KCHS and prevalence of hypertension by physical examination in KNHANES over six years was 8.4%p, with 12.9%p in 2010, 9.1%p in 2011, 8.8%p in 2012, 7.3%p in 2013, 5.1%p in 2014, and 7.4%p in 2015. The mean relative difference was 32.0%p.

The mean absolute difference between diabetes diagnosis rate by interview in KCHS and prevalence of diabetes by physical examination in KNHANES over six years was 1.9%p, with 2.1%p in 2010, 2.2%p in 2011, 1.5%p in 2012, 3.0%p in 2013, 1.5%p in 2014, and 1.1%p in 2015. The mean relative difference was 21.6%p.

### Comparison of time series stability of subgroup estimates between KCHS and KNHANES

**Figure 2.**
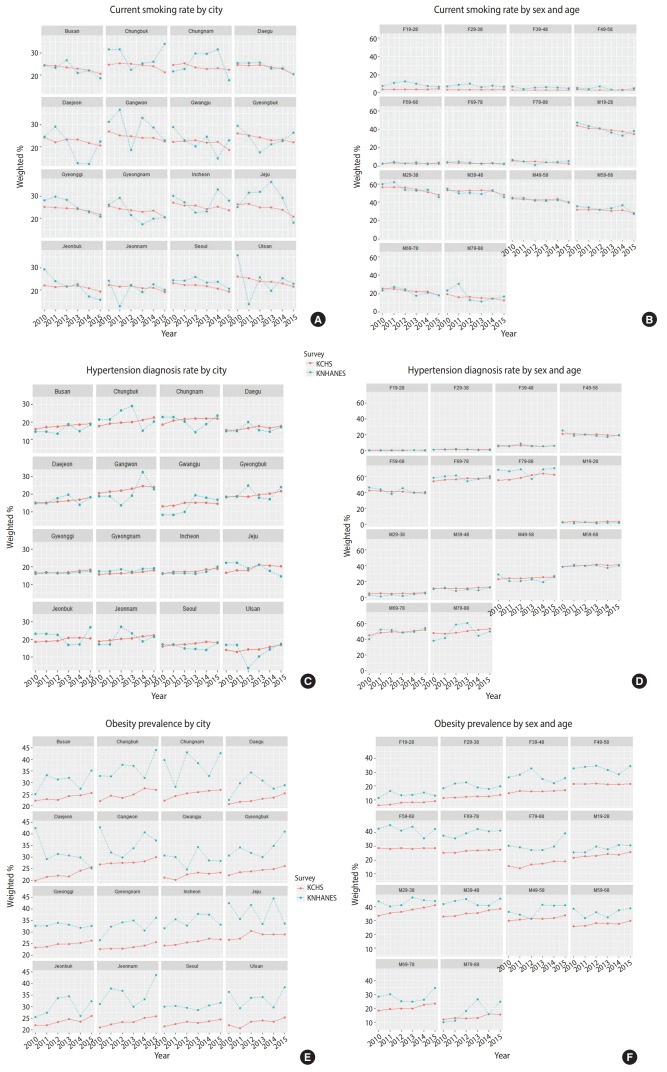
Time series trends (current smoking rate, hypertension diagnosis rate, and obesity prevalence) by city (A, C, and E) and by sex and age (B, D, and F) in Korea Community Health Survey (KCHS) and Korea National Health and Nutrition Examination Survey (KNHANES), 2010-2015. F, female; M, male.

Figure 2 shows the graph of time series trends by city as well as sex and age for current smoking rate, hypertension diagnosis rate and prevalence of obesity from 2010 to 2015. When divided by city, changes in current smoking rate estimates in KCHS were smaller than those in KNHANES in nearly all regions. However, when divided by sex and age, changes in estimates were similar between the two surveys. The trends for hypertension diagnosis rate were nearly identical to that of current smoking rate for both city as well as sex and age graphs. Regarding prevalence of obesity, KCHS computes the prevalence using self-reported height and weight while the KNHANES computes the prevalence using actually measured height and weight. For this reason, the difference of estimates is larger than that for current smoking rate or hypertension diagnosis rate. Similar to other variables, time series trends varied greatly for KNHANES in the city graph, but the variability was smaller in the sex and age graph.

Tables 3 shows the MSE values for each variable by city as well as by sex and age. The MSE values for city subgroups were 0.5, 0.3, and 0.4 in KCHS and 22.6, 13.0, and 16.4 in KNHANES, showing a higher time series stability in KCHS. The MSE values for sex and age subgroups were 0.6, 0.4, and 0.3 in KCHS and 7.0, 13.6, and 9.2 in KNHANES, also showing a higher time series stability in KCHS.

## DISCUSSION

This study compared the estimates for six variables measured based on a questionnaire in KCHS and eight variables measured based on a questionnaire or physical examination in KNHANES from 2010 to 2015 and divided them by city as well as sex and age to compare time series stability. With the exception of self-rated health, all estimates measured based on questionnaires, namely current smoking rate, monthly drinking rate, hypertension diagnosis rate and diabetes diagnosis rate, showed an absolute difference of less than 1.0%p and relative difference ranging from 1.99.0%p. For prevalence of obesity, hypertension diagnosis rate and prevalence of hypertension, diabetes diagnosis rate and prevalence of diabetes using questionnaire data in KCHS and physical examinations data in KNHANES, the absolute difference ranged from 1.9-8.4%p and relative difference ranged from 21.6-32.0%p, showing greater differences in the estimates compared to those measured based on questionnaire data. Time series stability by subgroup was higher for KCHS than KNHANES, and in KNHANES, time series stability for sex and age subgroups was greater than that for regional subgroups.

In both surveys, the difference of estimates for variables measured using questionnaire data was small, but there was a large difference of the estimates in self-rated health. Although both KCHS and KNHANES showed a declining trend in self-rated health, the difference between the two surveys was relatively large despite the fact that both surveys used the same question “how would you rate your health?” and both surveys collected this data using a questionnaire. Differences also varied across variables in the study conducted by Fahimi et al. [3] comparing BRFSS and other national surveys, where there were relatively small differences of current smoking rate and influenza vaccination rate in the past year in various subgroups but larger differences in prevalence of asthma and self-rated health. Particularly, the absolute difference of the percentage of “fair” or “poor” responses regarding self-rated health between surveys was greater (4.2%; relative difference 33.9%) than that for other variables [3]. Although the absolute difference of self-rated health between surveys in the present study (10.8%) was greater than that found by Fahimi et al. [3], the relative difference was similar at about 33.0%. Salomon et al. [7] compared self-rated health data among various national surveys from 1971 to 2007. In the said study, the estimates of self-rated health were compared among four surveys by dividing the participants by sex, age, race, and education. In general, the estimates differed greatly and showed inconsistent trends across surveys. In contrast, variables such as diabetes and BMI differed less and showed consistent time series trends across surveys. Based on these results, Salomon et al. [7] suggested that it is difficult to provide a simple explanation of the differences in the estimates of selfrated health across surveys and that self-rated health is not an appropriate variable for monitoring the health of different groups over time. In a study comparing the estimates among three national surveys by Li et al. [15], the absolute difference of current smoking rate, prevalence of obesity, prevalence of hypertension, and no health insurance rate ranged from 0.7-3.9%p. Particularly, the absolute difference of self-rated health ranged from 0.4-3.1%p, which suggests similar estimates across surveys, but the trends were inconsistent. As shown here, multiple studies report that self-rated health estimates differ greatly across surveys.

The difference of prevalence between data measured using questionnaires and physical examinations was greater than that between data measured using questionnaires in other studies as well. In a study that compared the prevalence of obesity between that measured using self-reported height and weight in KCHS and that computed using actually measured height and weight in KNHANES in 2010, the prevalence of obesity differed by 8.6%p and prevalence of overweight differed by 7.8%p. This was because overestimation of height increased with age while weight was underestimated in males in their 20s and 30s and females in their 20s to 40s in KCHS [16]. In a study investigating the effects of prevalence of obesity on diabetes according to method of survey in adults aged 45 years or older, the difference of obesity prevalence by questionnaire in KCHS and that by physical examination in KNHANES was 6.4%p and the difference of diabetes prevalence was 2.6%p [17]. Other study also reported that self-reported data and actually measured data differ particularly according to sex [18].

Time series trend by subgroup was more stable in KCHS than in KNHANES. This may be attributable to the fact that KCHS has about 40 times more subjects than KNHANES. Variability of estimates in the regional subgroups was high in KNHANES, particularly in regions with a small number of subjects (≤200). There was a tendency of higher variability of estimates in the 79-88 years group for males and females compared to other age groups in KNHANES, but this age group had fewer subjects (≤150) than other age groups. This suggests that time series stability of regional estimates is not ensured in KNHANES, so extra precaution should be taken when using regional analyses as evidence of policies or research.

Nevertheless, this study has a few limitations. We were able to compare the estimates of only a few items, so the findings cannot represent the overall differences of estimates between the two surveys. Furthermore, although both KCHS and KNHANES use complex samples, they differ in the number of subjects and sampling method. KCHS surveys about 220,000 people every year with 900 individuals in each of the 251 public health centers in 16 cities nationwide, which enables even distribution of samples throughout all regions in Korea [19]. In contrast, KNHANES has a relatively smaller sample of 10,000 people in 3,840 households in 192 districts every year using sex, age, living space, and education of head of household as implicit stratification standards, and it aims to compute yearly national statistics, which may cause uneven distribution of subjects across regions. Such differences of features limits direct comparison of the two surveys. Furthermore, KNHANES is an annual survey, whereas KCHS conducts a survey for three months from August to October, so there is a chance that the estimates may differ due to the difference in survey periods. In addition, KCHS collects data through in-person interviews, while KNHANES collects data either through interviews or selfreported questionnaire depending on the survey item. Quality management of interviewers also differs between the two surveys. For KCHS, interviewers are selected for each region in June, and they undergo short-term training [20]. For KNHANES on the other hand, eight specialists comprise a team for interview survey and physical examinations survey, two of whom take charge of health interview, and a total of four professional survey teams travel around the country for the survey [21]. As shown here, the two surveys differ in several aspects, and estimates can differ not only due to the differences in the number of subjects, sampling method, and data collection method but also due to subtle differences, such as those in the nature of health parameters, phrasing of questions, and order of questions [22,23]. Therefore, such minor differences should be meticulously reviewed when comparing different surveys [15]. When using KCHS and KNHANES data, the differences between the two surveys should be noted and either data should be selected or both should be used in supplementation depending on the purpose of analysis or policy.

Despite these limitations, this study clearly has strengths as well. Compared to similar Korean studies [16,17], we performed a more comprehensive comparison using six and eight variables in KCHS and KNHANES, respectively, over six years, and also analyzed the differences of estimates according to method of survey by comparing estimates taken from interviews and estimates taken from physical examinations data. Furthermore, we analyzed the time series stability of subgroup estimates and demonstrated that subgroup estimates might differ between the two surveys even with little differences in yearly estimates overall.

This study proposed the similarities and disparities between two major health surveys in Korea that are utilized in policies and research, and our findings would contribute to preventing errors that may occur by using only one set of data as the basis of policies or research. Recent inter-survey comparison studies have expanded the scope of comparison to surveys across countries [24]. In the future, studies should compare Korean surveys with foreign surveys to lay a foundation to compare and share international policies.

## Figures and Tables

**Table 1.** Participants aged 19 years or older in the 2010-2015 Korea Community Health Survey (KCHS) and Korea National Health and Nutrition Examination Survey (KNHANES)

Year	KCHS		KNHANES		Ratio (KCHS/ KNHANES)
	n	Weighted n	n	Weighted n	
2010	229,126	38,999,317	6,254	38,226,440	1.02
2011	229,186	39,904,832	6,027	39,050,481	1.02
2012	228,899	40,363,395	5,611	39,674,018	1.02
2013	228,764	40,781,906	5,362	40,164,541	1.02
2014	228,695	41,143,286	5,040	40,767,231	1.01
2015	228,558	41,554,658	5,571	41,176,539	1.01

**Table 2.** Range of difference, mean of difference and relative difference between the estimates of Korea Community Health Survey (KCHS) and Korea National Health and Nutrition Examination Survey (KNHANES), 2010-2015

Variables	Range of difference	Mean difference^1^	95% confidence interval	Relative difference^2^
Self-rated health	9.2-1.4	10.8	9.1, 12.5	33.0
Current smoking rate	0.1-2.3	1.2	-0.3, 2.8	4.9
Monthly drinking rate	0.4-2.2	1.1	-0.6, 3.0	1.9
Hypertension diagnosis rate	0.0-2.1	0.8	-0.6, 2.2	4.6
Diabetes diagnosis rate	0.1-1.0	0.6	-0.2, 1.3	9.0
Obesity prevalence (self-reported height & weight, KCHS) vs. obesity prevalence (actually measured height & weight, KNHANES)	6.6-8.9	8.3	6.7, 9.9	25.6
Hypertension diagnosis rate (interview, KCHS) vs. prevalence of hypertension (physical exam, KNHANES)	5.1-12.9	8.4	6.7, 10.2	32.0
Diabetes diagnosis rate (interview, KCHS) vs. prevalence of diabetes (physical exam, KNHANES)	1.1-3.0	1.9	0.9, 2.9	21.6

Values are presented as %.

1 Mean difference (absolute difference) is the average estimate of the differences for each year.

2 Relative difference = mean difference (%, absolute difference) divided by average estimate(%) of 6-year of KNHANES.

**Table 3.** Mean square error by city, sex and age in Korea Community Health Survey (KCHS) and Korea National Health and Nutrition Examination Survey (KNHANES), 2010-2015

Variables	KCHS			KNHANES		
	Current smoking rate	Hypertension diagnosis rate	Obesity prevalence	Current smoking rate	Hypertension diagnosis rate	Obesity prevalence
City						
Busan	0.2	0.0	0.1	4.4	4.0	13.7
Chungbuk	1.0	0.1	0.9	25.8	34.4	15.8
Chungnam	0.4	0.6	0.3	39.2	15.1	40.6
Daegu	0.7	0.3	0.1	1.0	6.3	17.6
Daejeon	0.9	0.2	0.5	38.7	6.1	16.5
Gangwon	0.2	0.2	0.3	46.7	35.5	32.2
Gwangju	1.6	0.5	0.6	18.6	8.8	12.5
Gyeongbuk	0.1	0.2	0.1	20.3	13.4	10.6
Gyeonggi	0.2	0.1	0.1	2.4	0.2	0.6
Gyeongnam	0.6	0.1	0.3	11.3	1.9	8.5
Incheon	0.4	0.1	0.1	20.3	2.1	7.2
Jeju	1.0	1.0	1.6	46.7	3.7	27.1
Jeonbuk	0.4	0.2	0.5	2.8	19.5	17.0
Jeonnam	0.2	0.0	0.1	20.0	21.4	25.3
Seoul	0.1	0.1	0.1	1.8	3.1	1.2
Ulsan	0.1	0.5	0.8	61.1	33.0	15.6
Mean	0.5	0.3	0.4	22.6	13.0	16.4
Sex						
Female						
19-28	0.0	0.0	0.1	5.9	0.0	3.8
29-38	0.0	0.0	0.1	2.1	0.3	4.4
39-48	0.0	0.1	0.2	1.0	2.4	13.5
49-58	0.1	0.1	0.1	3.1	5.8	5.8
59-68	0.0	0.1	0.1	1.1	9.7	11.1
69-78	0.0	0.5	0.1	0.4	8.7	3.1
79-88	0.1	2.1	1.2	3.7	30.8	17.9
Male						
19-28	0.3	0.3	0.3	9.4	9.4	1.8
29-38	1.8	0.1	0.1	8.1	1.8	5.8
39-48	2.0	0.2	0.3	8.8	4.4	6.6
49-58	0.9	0.1	0.3	0.9	19.6	12.1
59-68	0.9	0.5	0.6	10.7	2.5	10.8
69-78	0.8	1.1	0.7	7.5	18.9	16.1
79-88	0.8	1.0	0.8	41.7	89.7	25.6
Mean	0.6	0.4	0.3	7.0	13.6	9.2

## SUPPLEMENTARY MATERIALS

Korean version is available at http://www.e-epih.org/).
