# Supplementary material for: Comparison of estimates and time series stability of Korea Community Health Survey and Korea National Health and Nutrition Examination Survey
Source: Epidemiol Health. 2019 Apr 7;41:e2019012. doi: 10.4178/epih.e2019012 (PMC6533555; doi:10.4178/epih.e2019012)
Supplement: Supplementary file 1 [file epih-41-e2019012-supplementary.pdf]

Volume: 41, Article ID: e2019012
https://doi.org/10.4178/epih.e2019012

지역사회건강조사와 국민건강영양조사의 추정치 및 시계열 안정성 비교

First author: Ji Son Ki, ORCID: 0000-0002-8938-5546

College of Nursing, Seoul National University, Korea

Corresponding author: Ho Kim, ORCID: 0000-0001-7472-3752

Graduate School of Public Health, Seoul National University,
1 kwanak-ro Kwanak-gu, Seoul 08826, Korea
Telephone: + (82) 2 880-2701
E-mail address: hokim@snu.ac.kr

초록

연구목적: 우리나라 국민의 건강상태를 파악하기 위한 대표적인 조사는 질병관리본부에서 실시하고 있는 지역사회건강조사와 국민건강영양조사가 있다. 두 조사는 목표 모집단이 같고 공통된 문항이 있으므로 비교 가능하며 두 조사의 자료가 다양한 분석에 쓰이고 있기 때문에 추정치 비교를 통해 유사점과 차이점을 파악한다면 자료의 적절한 활용에 지표가 될 수 있을 것이다. 그러므로 지역사회건강조사 6개 변수와 국민건강영양조사 8개 변수의 6년간의 추정치를 비교해 보고, 이를 시도별, 성연령별로 나누어 하위그룹 추정치의 시계열 안정성을 비교해 보고자 한다.

대상 및 방법 : 지역사회건강조사와 국민건강영양조사의 2010년부터 2015년까지 만 19세 이상 성인 자료를 이용하여 양호한 주관적 건강수준 인지율, 현재흡연율, 월간음주율, 고혈압 의사진단 경험률, 당뇨병 의사진단 경험률, 비만 유병율, 고혈압 유병률, 당뇨병 유병률의 추정치 차이와 95% 신뢰구간을 분석하고, 변수별로 시도 및 성연령별 하위그룹으로 나누어 추정치의 시계열 안정성을 평균제곱오차로 평가해 본다.

결과: 양호한 주관적 건강수준 인지율을 제외하면 두 조사 모두 설문 자료를 이용한 현재흡연율, 월간음주율, 고혈압 의사진단 경험율, 당뇨병 의사진단 경험율은 1%P내외의 차이를 보였다. 국민건강영양조사의 검진 자료를 이용한 비만 유병율,

고혈압 유병율, 당뇨병 유병율 추정치의 경우 각각 1.9~8.4%P차이를 보여 설문 자료를 이용한 추정치의 차이보다 컸다. 하위그룹별 시계열 안정성은 지역사회건강조사가 국민건강영양조사보다 더 안정적인 것으로 나타났다.

고찰: 지역사회건강조사와 국민건강영양조사 자료 이용 시 두 자료의 다양한 차이를 염두에 두고 분석이나 정책의 목적에 따라 자료를 선택하고 상호 보완하여 사용해야 할 것이다.

Keywords: 지역사회건강조사, 국민건강영양조사, 추정치, 비교, 시계열 안정성

Volume: 41, Article ID: e2019012
https://doi.org/10.4178/epih.e2019012

서론

공중 보건에 관한 설문조사는 현대 역학의 기초이며, 국민의 건강을 증진하고 질병을 예방하기 위한 정책 및 프로그램의 수립을 위해 시행된다[1]. 또한 주기적으로 시행되어지며 정확한 보건 조사 자료는 보건문제의 범위와 추세를 이해하는데 매우 유용하다 [2]. 특히 연구자와 정책입안자는 이러한 설문자료를 바탕으로 현재와 미래의 보건문제를 이해하고 예측할 수 있으며 한정된 자원이 효과적으로 쓰일 수 있도록 할 수 있다[3].

우리나라 공공보건, 복지분야의 대표적인 설문조사는 한국질병관리본부에서 주관하고 있는 지역사회건강조사와 국민건강영양조사가 있다. 지역사회건강조사는 우리나라 지역보건의료계획 수립에 필요한 시 · 군 · 구 단위 건강통계를 산출하여 지역간 비교를 가능하게 하며 지역보건사업의 지표가 되고 있고[4], 국민건강영양조사는 국민의 건강수준, 건강관련 의식 및 행태, 식품 및 영양섭취 실태에 대한 국가단위의 통계를 산출하여 국민건강증진종합계획의 목표설정 및 평가지표로 활용되고 있으며, 세계보건기구(World Health Organization)와 경제협력개발기구(Organisation for Economic Cooperation and Development) 등에서 요청하는 흡연, 음주, 신체활동, 비만 관련 전국 통계자료를 제공하고 있다[5]. 이 두 조사는 공통적으로 대한민국 국민을 목표 모집단으로 하고 복합표본설계로 대한민국 전지역을 조사범위에 포함하고 있다. 또한 서로 다른 목적을 가지고 있긴 하지만 설문의 많은 항목이 중복된다.

외국의 경우에는 조사의 목적은 다르지만 중복된 설문이 있는 경우 추정치를 비교함으로써 설문조사의 타당성을 평가하는 연구가 지속적으로 이루어지고 있다. 설문조사 추정치의 타당성은 모집단 전수를 조사하는 방법이 가장 정확하나 이는 현실적으로 불가능하므로 의료기록이나 다른 설문조사 추정치와 비교함으로써 평가할 수 있다[6]. 미국의 경우 주관적 건강수준에 대해 국가단위 보건 설문조사인 Behavioral

Risk Factor Surveillance System, Current Population Survey, National Health and Nutrition Examination Survey, National Health Interview Survey 등 총 4개 조사의 추정치를 비교하는 연구[7], 폭음율에 대해 Behavioral Risk Factor Surveillance System와 National Survey on Drug Use and Health의 추정치를 비교하는 연구[8], 비만율에 대해 Behavioral Risk Factor Surveillance System와 National Health and Nutrition Examination Survey의 추정치를 비교하는 연구[9]등 다수의 연구가 이루어졌다.

그러나 우리나라는 아직까지 국가단위 설문조사 추정치에 대한 비교 연구가 드물다. 매년 실시하는 보건분야 국가단위 설문조사가 지역사회건강조사와 국민건강영양조사 외에는 거의 없고 두 조사의 근본적인 목적이 달라 어느 한 조사를 기준으로 두고 다른 조사의 타당성을 평가하는 것은 논란의 여지가 있기 때문이다. 하지만 지역사회건강영양조사나 국민건강영양조사 모두 정책개발이나 연구의 자료로 활발히 활용되고 있고 두 조사의 본래 목적과 다르게 다양한 분석에 쓰이고 있기 때문에 [10-13] 추정치 비교를 통해 유사점과 차이점을 파악한다면 자료의 적절한 활용에 지표가 될 수 있을 것이다. 정책입안자 혹은 연구자가 두 조사의 자료를 활용할 때 개인의 편의가 아닌 자료의 특성과 분석의 목적에 따라 자료를 선택하거나 두 자료의 단점을 적절히 보완하여 활용한다면 현재의 보건문제를 더욱 정확히 파악하는데 도움이 될 수 있을 것이다.

그러므로 본 연구에서는 국가 보건의 중요 통계를 산출하는 기초가 되는 조사인 지역사회건강조사와 국민건강영양조사의 추정치를 비교하고자 한다. 그 방법으로 지역사회건강조사의 설문 6개 변수와 국민건강영양조사의 설문과 검진을 포함한 8개 변수에 대해 6년간의 조사 추정치를 비교분석하고 나아가 시도별, 성연령별 하위그룹 추정치의 시계열 안정성을 살펴봄으로써 두 조사의 추정치를 더 자세히 비교해 보고자

Volume: 41, Article ID: e2019012
https://doi.org/10.4178/epih.e2019012

하였다.

연구대상 및 방법

1. 연구자료

연구자료로 2010년부터 2015년까지 국내에서 시행된 지역사회건강조사와 국민건강영양조사 자료를 이용하였다. 두 조사 모두 한국질병관리본부에서 주관하고 있는 조사로 지역사회건강조사는 전국 만19세 이상 성인을 대상으로 표본가구로 선정된 가구의 가구원 모두에게 면접설문조사를 시행한다. 시 · 군 · 구당 900명을 조사하여 매년 약 22만명을 조사하고 있으며 표본추출은 통 · 반/리 내 주택유형별 가구 수를 기준으로 가구 수 크기를 고려하여 추출확률이 비례하도록 추출하고 계통 추출법으로 2차 표본 가구를 선정한다[4]. 국민건강영양조사는 전국 만1세 이상 표본가구로 선정된 가구원을 대상으로 매년 약 1만명에게 조사를 시행한다. 지역사회건강조사와 마찬가지로 시도, 동읍면, 주택유형을 층화변수로 사용한 복합표본설계로 표본을 추출하며 매년 192개 조사구의 총 3840가구를 선정하여 대상자의 생애주기별 특성에 따라 소아(1세~11세), 청소년(12세~18세), 성인(19세)이상으로 나누어, 각기 특성에 맞는 조사항목을 적용하고 모든 대상자에 대해 건강설문조사, 검진조사, 영양조사를 실시한다[5].

(Table 1)

본 연구에서는 대상을 만 19세 이상으로 한정하였고 조사 대상자수는 Table 1과 같다. 지역사회건강조사와 국민건강영양조사의 조사 대상자 수가 매년 약40배 정도의 차이를

보이지만 복합표본설계된 자료임을 반영하여 표본의 목표 모집단인 대한민국 국민을 대표하도록 가중치를 고려하여 분석하면 가중치가 부여된 대상자의 수는 두 조사가 매년 비슷한 수치를 보인다. 두 조사의 비는 2010~2013년까지는 2%의 차이를 보이고 2014~2015년까지는 1%의 차이를 보여 두 조사의 가중치가 부여된 대상자 수는 6년간 평균 2% 정도의 차이를 보인다.

가중치가 부여된 대상자를 시도별, 성연령별 하위그룹으로 나누면 2015년 현재흡연율 변수 기준으로 평균 9%, 5%의 차이를 보인다. 이러한 차이는 연도별 변수별로 상이하며 무응답으로 인해 발생한다. 본 연구에서는 이러한 차이도 자료 특성의 한 부분으로 간주하여 연령표준화를 하지 않고 그대로 분석하였다.

2. 변수의 정의

편향(bias)을 줄이기 위해 두 조사의 질문이 되도록 동일한 변수를 택하여 분석하였다. 지역사회건강조사는 양호한 주관적 건강수준 인지율, 현재흡연율, 월간음주율, 고혈압 평생 의사진단 경험률, 당뇨병 평생 의사진단 경험률, 비만 유병률로 모두 6개의 변수를 사용하였다. 국민건강영양조사는 양호한 주관적 건강수준 인지율, 현재흡연율, 월간음주율, 고혈압 의사진단 경험률, 당뇨병 의사진단 경험률, 비만 유병률, 고혈압 유병률, 당뇨병 유병률로 모두 8개의 변수를 사용하였다. 국민건강영양조사의 비만 유병률, 고혈압 유병률, 당뇨병 유병률의 경우 검진자료를 이용하여 분석하였고 나머지 변수는 두 조사 모두 설문자료를 이용하여 분석하였다.

양호한 주관적 건강수준 인지율은 평소에 본인의 건강을 매우좋음, 좋음으로 답한 비율이며, 현재 흡연율은 지금까지 살아오는 동안 5갑(100개비)이상의 담배를 피우고 현재 흡연상태를 매일 피우거나 가끔 피운다고 답한 비율, 월간 음주율은 지금까지 살아오면서 1잔 이상의 술을 마신 적이 있으며 술을 한달에 한번 이상 마신다고 답한

Volume: 41, Article ID: e2019012
https://doi.org/10.4178/epih.e2019012

비율을 말한다. 지역사회건강조사의 고혈압 평생 의사진단 경험률은 의사에게 고혈압 진단을 받은 적이 있다고 답한 비율, 당뇨병 평생 의사진단 경험률은 의사에게 당뇨병 진단을 받은 적이 있다고 답한 비율을 말한다. 국민건강영양조사의 고혈압 의사진단 경험률은 고혈압 의사진단 여부를 묻는 질문에 있음이라고 답한 비율로 정의하여 산출하였으며 당뇨병 의사진단 경험률도 당뇨병 의사진단 여부를 묻는 질문에 있음이라도 답한 비율을 정의하여 산출하였다.

지역사회건강조사는 설문을 통해 자가보고 한 신장과 체중으로, 국민건강영양조사는 검진시 신체계측한 신장과 체중으로 각각 체질량지수(Body Mass Index; BMI)를 계산하였다. 세계보건기구의 아시아-태평양 지역기준에 따라 25Kg/m^2 이상을 비만으로 정의하고[14], 이의 비율을 비교하였다. 국민건강영양조사의 고혈압 유병률은 검진 조사에서 3회의 혈압측정으로 수축기혈압이 140mmHg 이상 또는 이완기혈압이 90mmHg 이상 또는 고혈압 약물을 복용한 경우 고혈압 유병으로 정의하고 이의 비율을 지역사회건강조사의 고혈압 평생 의사진단 경험율과 비교하였다. 국민건강영양조사의 당뇨병 유병률은 검진 조사에서 8시간이상 공복자 중 공복혈당이 126mg/dl 이상이거나, 의사진단을 받았거나 혈당강하제 복용하거나, 인슐린주사를 투여받고 있는 사람을 당뇨병 유병으로 정의하고 이의 비율을 지역사회건강조사의 당뇨병 평생 의사진단 경험율과 비교하였다. 고혈압이나 당뇨병의 경우 완치보다는 조절이 가능한 만성질환이기때문에 한번이라도 진단을 받은 적이 있다고 답한 경우 질병이 있다고 간주하여 검진이라는 객관적인 방법으로 측정한 병의 유무와의 비교가 설문 조사와 검진 조사의 비교를 상당부분 대체할 수 있다고 보았다.

3. 통계분석

두 조사 모두 복합표본설계된 자료임을 고려하여 추정치 산출 시 가중치, 층화변수, 집락변수를 고려하였다. 추정치 비교는 변수별 연도별 추정치를 이용하여 두 조사의 절대적 차이와 차이의 95% 신뢰한계(95% confidence limit), 절대적 차이와 국민건강영양조사의 평균 추정치의 비로 상대적 차이를 구하였다. 절대적 차이는 추정치 차이의 절대값이고 상대적 차이는 평균 추정치에서 절대적 차이가 차지하는 비율로 추정치가 작은 변수는 절대적 차이도 작은 경향이 있어 상대적 차이를 살펴봄으로써 추정치 크기의 영향을 받지 않는 좀 더 명확한 차이를 알 수 있다.

시계열 안정성은 국민건강영양조사의 경우 제6기(2013~2015년)까지는 세종시를 분리하여 조사하지 않았으므로 세종시를 제외한 16개 시도별 하위그룹과 남녀 19세부터 88세까지 10세 단위로 나눈 14개 성연령별 하위그룹으로 나누어 비교하였다. 각 변수의 하위그룹별 6년간의 추정치로 단순선형회귀직선을 구하고 이 직선에 대한 추정치의 평균제곱오차(MSE: Mean square error)로 추정치의 변동성을 평가하였다. 예를 들어 6년동안 추정치의 추세에 대한 변동이 크지 않은 경우 평균제곱오차가 작아지게 되고 이는 시계열 안정성이 높다고 평가할 수 있다. 모든 분석은 SAS(버전9.4)와 R(버전3.4.4)를 이용하여 수행되었다.

연구결과

1. 지역사회건강조사와 국민건강영양조사의 변수별 추정치 비교

2010년부터 2015년까지 6년간 두 조사의 변수별 추정치를 비교한 결과는 다음과 같다(Figure1 & Table 2). 양호한 주관적 건강수준 인지율(Self-rated health)의 절대적 차이는 2010년 10.5%P, 2011년 9.2%P, 2012년 11.4%P, 2013년 9.6%P,

2014년 9.9%P, 2015년 14.0%P로 평균 10.8%P의 차이를 보였고 상대적 차이는 평균 33%P였다. 이는 다른 변수에 비해 큰 차이였다.

현재흡연율(Current smoking rate)의 절대적 차이는 2010년 2.3%P, 2011년 2.3%P, 2012년 1.3%P, 2013년 0.1%P, 2014년 0.6%P, 2015년 0.6%P로 평균 1.2%P의 차이를 보였다. 상대적 차이는 4.9%P였다.

월간음주율(Monthly drinking rate)의 절대적 차이는 2010년 2.2%P, 2011년 0.7%P, 2012년 1.1%P, 2013년 0.4%P, 2014년 1.4%P, 2015년 1.0%P로 평균 1.1%P의 차이를 보였다. 상대적 차이는 1.9%P였다.

지역사회건강조사의 고혈압 평생 의사진단 경험률(Hypertension diagnosis rate)과 국민건강영양조사의 고혈압 의사진단 경험률의 절대적 차이는 2010년 0.8%P, 2011년 0.3%P, 2012년 0.3%P, 2013년 1.1%P, 2014년 2.1%P, 2015년 0.0%P로 평균 0.8%P의 차이를 보였다. 상대적 차이는 평균 4.6%P였다.

지역사회건강조사의 당뇨병 평생 의사진단 경험률(Diabetes diagnosis rate)과 국민건강영양조사의 당뇨병 의사진단 경험률의 절대적 차이는 2010년 0.1%P, 2011년 0.2%P, 2012년 0.6%P, 2013년 0.8%P, 2014년 1.0%P, 2015년 0.7%P로 평균 0.6%P의 차이를 보였다. 상대적 차이는 평균 9.0%P였다.

지역사회건강조사의 자가보고한 신장과 체중으로 계산한 비만 유병률(Obesity prevalence by self-reported height & weight)과 국민건강영양조사의 실측한 신장과 체중으로 계산한 비만 유병률(Obesity prevalence by actually measured height & weight)의 절대적 차이는 2010년 8.9%P, 2011년 8.7%P, 2012년 8.8%P, 2013년 8.3%P, 2014년 6.6%P, 2015년 8.2%P로 평균 8.3%P의 차이를 보였다. 상대적 차이는 평균 25.6%P였다.

지역사회건강조사 설문의 고혈압 평생 의사진단 경험률(Hypertension diagnosis rate

by interview)과 국민건강영양조사 검진의 고혈압 유병률(Prevalence of hypertension by physical exam)의 차이는 2010년 12.9%P, 2011년 9.1%P, 2012년 8.8%P, 2013년 7.3%P, 2014년 5.1%P, 2015년 7.4%P로 평균 8.4%P의 차이를 보였다. 상대적 차이는 평균 32.0%P였다.

지역사회건강조사 설문의 당뇨병 평생 의사진단 경험률(Diabetes diagnosis rate by interview)과 국민건강영양조사 검진의 당뇨병 유병률(Prevalence of diabetes by physical exam)의 차이는 2010년 2.1%P, 2011년 2.2%P, 2012년 1.5%P, 2013년 3.0%P, 2014년 1.5%P, 2015년 1.1%P로 평균 1.9%P의 차이를 보였다. 상대적 차이는 평균 21.6%P였다.

(Figure 1 & Table 2)

2. 지역사회건강조사와 국민건강영양조사의 하위그룹별 추정치의 시계열안정성 비교

Figure 2는 2010년부터 2015년까지 현재 흡연율, 지역사회건강조사의 고혈압 평생 의사진단 경험율과 국민건강영양조사의 의사진단 경험률, 비만 유병률에 대해 두 조사의 추정치를 시도별, 성연령별 하위그룹으로 나누어 시계열 추세 그래프로 나타낸 것이다. 현재 흡연율은 시도별로 나누어 보았을 때 거의 모든 지역에서 지역사회건강조사 추정치의 변화가 국민건강영양조사보다 작은 것을 볼 수 있다. 그러나 성연령별로 나누어 보면 두 조사 추정치의 변화가 비슷한 것을 확인할 수 있다. 고혈압 의사진단 경험율의 경우도 시도별, 성연령별 그래프 모두 현재 흡연율과 거의 동일한 패턴을 보이고 있다. 비만 유병률의 경우 지역사회건강조사는 자가보고한 신장과 체중으로 산출한 반면, 국민건강영양조사는 실측한 신장과 체중으로 산출하여 현재 흡연율이나 고혈압 의사진단 경험율

보다 추정치의 차이가 크며 이는 변수별 추정치 비교에서도 확인된 바 있었다. 시계열 추세는 다른 변수와 마찬가지로 시도별 그래프에서는 국민건강영양조사의 변동이 크게 나타났으며 성연령별 그래프에서는 그 변동이 줄어든 것을 확인할 수 있다.

Table 3는 각 변수의 시도별 성연령별 하위그룹의 평균제곱오차(Mean Square Error; MSE)를 보여준다. 시도별 하위그룹의 변수별 평균제곱오차는 지역사회건강조사는 평균 0.5, 0.3, 0.4으로 나타났고 국민건강영양조사는 평균 22.6, 13.0, 16.4로 시도별 추정치의 시계열 안정성은 지역사회건강조사가 높은 것으로 나타났다. 성연령별 하위그룹의 변수별 평균제곱오차는 지역사회건강조사는 평균 0.6, 0.4, 0.3으로 나타났고 국민건강영양조사는 평균 7.0, 13.6, 9.2로 성연령별 추정치의 시계열 안정성도 지역사회건강조사가 높은 것으로 나타났다.

(Figure 2 & Table 3)

고찰

본 연구는 2010년부터 2015년까지 지역사회건강조사의 설문을 이용한 6개 변수와 국민건강영양조사의 설문과 검진을 이용한 8개 변수의 추정치를 비교하고 시도별, 성연령별 하위그룹으로 나누어 추정치의 시계열 안정성을 비교하였다. 양호한 주관적 건강수준 인지율을 제외하면 두 조사 모두 설문 자료를 이용한 현재흡연율, 월간음주율, 지역사회건강조사의 고혈압 평생 의사진단 경험률과 국민건강영양조사의 고혈압 의사진단 경험률, 지역사회건강조사의 당뇨병 평생 의사진단 경험률과 국민건강영양조사의 당뇨병 의사진단 경험률은 1%P내외의 절대적 차이와 1.9~9%P의 상대적 차이를 보였다. 지역사회건강조사의 설문 자료와 국민건강영양조사의 검진 자료를 이용한 비만 유병률, 고혈압 평생 의사진단 경험률과 고혈압 유병률, 당뇨병

평생 의사진단 경험률과 당뇨병 유병률 추정치의 경우 각각 1.9~8.4%P의 절대적 차이와 21.6~32.0%P의 상대적 차이를 보여 설문 자료를 이용한 추정치의 차이보다 컸다. 하위그룹별 시계열 안정성은 지역사회건강조사가 국민건강영양조사보다 더 안정적인 것으로 나타났으며 국민건강영양조사는 성연령별 추정치의 시계열 안정성이 시도별 추정치의 시계열 안정성보다 더 높은 것으로 나타났다.

두 조사 모두 설문자료를 이용한 변수의 경우 대체로 추정치의 차이가 매우 작았지만 양호한 주관적 건강수준 인지율은 그 차이가 컸다. 양호한 주관적 건강수준 인지율의 경우 지역사회건강조사와 국민건강영양조사 모두 감소하는 추세는 동일하였지만 비교적 큰 차이를 보였는데 '평소에 본인의 건강은 어떻다고 생각합니까' 라는 동일 문항을 사용하고 공통적으로 설문을 이용한 자료수집이라는 공통점에도 불구하고 이러한 차이를 보였다. Fahimi et al. 의 Behavioral Risk Factor Surveillance System과 다른 국가단위 조사를 비교한 연구에서도 변수에 따른 차이가 다양했는데 평생 흡연 경험률, 지난 1년동안 인플루엔자 백신을 접종한 경험률 등은 다양한 하위그룹에서 비교적 작은 차이를 보인 반면 천식 유병률, 주관적 건강수준 인지율 등은 큰 차이를 보였다. 특히 주관적 건강수준 인지율의 경우 보통 혹은 나쁨으로 답한 분율의 조사간 차이가 절대적 차이 4.16%P(상대적 차이 33.9%)로 다른 변수들 보다 큰 차이를 보였다[3]. 본 연구의 양호한 주관적 건강수준 인지율의 조사간 절대적 차이가 10.8%로 Fahimi et al.의 연구의 절대적 차이보다 크지만 상대적 차이는 33.0%로 비슷한 결과를 보였다. Salomon et al. 은 주관적 건강수준 인지율에 관해 1971년부터 2007년까지 국가단위 설문조사 자료를 비교 연구 하였다. 이 연구에서는 성, 연령, 인종, 교육 수준 등에 따라 하위집단으로 나누어 주관적 건강수준에 대한 4개 조사의 추정치 비교하였는데 대체적으로 조사간 추정치 차이가 컸고 트렌드 또한 일치하지 않는 경향을 보였다. 그에 반해 당뇨병과 BMI와 같은 변수의 경우에는 조사간 추정치의 차이가 적었고 시간의

흐름에 따른 트렌드가 일치함을 보였다. 이러한 결과에 대해 Salomon et al.은 조사간 주관적 건강수준 추정치의 차이를 단순히 설명하기는 어려우며 주관적 건강수준 인지율이 시간의 흐름에 따른 집단의 건강을 모니터하기에는 적절하지 않은 변수라는 해석을 덧붙이고 있다[7]. Chaoyang et al.의 3개의 국가단위 설문조사의 추정치를 비교한 연구에서는 현재 흡연율, 비만 유병률, 고혈압 유병률, 건강보험 미가입율은 0.7~3.9%P의 절대적 차이를 보였으며 주관적 건강수준 인지율의 경우 절대적 차이가 0.4%~3.1%P로 차이의 크기는 비슷하였으나 추세는 일관되지 않은 경향성을 보였다[15]. 이와 같이 주관적 건강수준 인지율의 경우 조사마다 추정치의 차이가 큼을 다수의 연구가 보고하고 있다.

설문조사와 검진조사의 유병률의 차이는 다른 연구에서도 설문조사간 차이보다 컸다. 만 20세 이상을 대상으로 2010년 지역사회건강조사의 자가보고한 신장과 체중으로 산출한 비만 유병률과 2010년 국민건강영양조사의 실측한 신장과 체중으로 산출한 비만 유병률을 비교한 연구에서 비만 유병률은 8.6%P, 과체중은 7.8%P 차이를 보였다. 이는 지역사회건강조사에서 신장의 과대평가가 나이가 증가할수록 컸고 20~30대 남자, 20~40대 여자에서 체중이 저평가되어 나타난 결과이다[16]. 45세 이상을 대상으로 조사 방법에 따른 비만 유병률이 당뇨병에 미치는 영향에 관한 연구에서도 지역사회건강조사의 설문과 국민건강영양조사의 검진에 의한 비만 유병률의 차이가 6.4%P, 당뇨병은 2.6%P의 차이를 보였다[17]. 그 외 연구에서도 자가보고 된 자료와 검진을 이용한 실측 자료는 특히 성별에 따라 차이가 난다고 보고된 바 있다[18].

하위그룹별 시계열 변동은 지역사회건강조사가 국민건강영양조사보다 더 안정적이었다. 이는 지역사회건강조사가 국민건강영양조사보다 조사 표본 대상자수가 40배 정도 많은 점에서 기인한다고 생각된다. 특히 시도별 하위그룹 추정치의 변동이 국민건강영양조사에서 크게 나타났는데 시도별 하위그룹으로 나눌 경우 조사대상자수가

적은 지역(200명 이하)에서 추정치의 변동이 크게 나타나는 경향을 보였다. 성연령별 하위그룹 추정치 변동도 국민건강영양조사의 남녀 79~88세 그룹이 다른 그룹에 비해 크게 나타나는 경향이 있는데 조사대상자수(150명 이하)가 다른 그룹에 비해 적었다. 이는 국민건강영양조사의 지역별 추정치에서 시계열 안정성이 확보되지 않으므로 지역별 분석을 연구분석이나 정책의 근거로 쓸 때에는 특히 주의를 기울여야 함을 시사하고 있다.

그럼에도 불구하고 본 연구에는 몇 가지 제한점이 있다. 두 조사의 추정치를 비교하는데 있어 매우 많은 문항 중 몇 개의 항목만을 비교하였고 이것은 이 두 조사의 일부분만을 비교한 것이므로 전제적인 추정치 차이를 대변할 수 없을 것이다. 그리고 지역사회건강조사와 국민건강영양조사 모두 복합표본설계되어 있는 자료이지만 조사대상수와 표본추출 방법의 차이점이 존재한다. 지역사회건강조사의 경우 전국 16개 시도 251개 보건소에서 지점과 개인을 추출 단위로 하여 900명씩 매년 약22만명을 조사하여 조사대상자 수가 많고 전지역이 고르게 표본 추출이 되는 반면[19], 국민건강영양조사의 경우 성별, 연령, 주거면적 비율, 가구주 학력비율 등을 내재적 층화기준으로 사용하며 조사구와 가구를 추출 단위로 연간 192조사구, 3840가구, 1만명을 조사하여 대상자 수가 상대적으로 적고 1년 단위의 전국 통계 산출을 목적으로 하기에 지역별로 조사대상자의 분포가 고르지 않을 수 있어 비교에 한계가 있을 수 있다. 또한 국민건강영양조사의 경우 연중 조사이며 지역사회건강조사는 8월~10월까지 3개월동안 조사를 실시하기 때문에 조사기간의 차이에 따른 추정치의 차이가 있을 수 있음을 배제할 수 없다. 같은 설문조사라 할 지라도 지역사회건강조사의 경우에는 조사원의 질문에 답하는 1:1 면접조사이며 국민건강영양조사는 문항에 따라 면접조사 또는 자기기입 방법으로 조사하여 조사방법에 차이가 있다. 두 조사의 조사원 질 관리도 차이를 보인다. 지역사회건강조사의 경우 매년 6월 지역별로 조사원을 선발하여 단기간

교육 후 조사를 실시하는 반면[20], 국민건강영양조사는 설문조사와 검진조사를 위해 8명의 조사원이 한 팀을 이루며 이 중 2명이 건강설문조사를 전담하고 총 4팀의 전문조사수행팀이 전국을 돌며 조사를 수행한다[21]. 이렇듯 두 조사는 다양한 차이가 존재하고, 조사 대상자 수, 대상자 선정방법, 자료수집방법뿐만 아니라 건강지표의 성격, 질문의 형식, 질문의 순서 등의 미세한 차이에 의해서도 추정치의 차이가 발생할 수 있다[22, 23]. 따라서 조사간 비교 시 이러한 사소한 차이까지도 면밀히 검토되어야 한다[15]. 지역사회건강조사와 국민건강영양조사 자료 이용시 두 자료의 다양한 차이를 염두에 두고 분석이나 정책의 목적에 따라 자료를 선택하고 상호 보완하여 사용해야 할 것이다.

이와 같이 본 연구의 한계점도 있지만 장점 또한 분명하다. 지역사회건강조사와 국민건강영양조사의 6년치 자료와 각각 6개, 8개 변수를 이용하여 기존 국내 연구[16, 17]에 비해 포괄적인 비교를 하였고 설문 추정치와 검진 추정치를 동시에 비교함으로써 조사 방법의 차이에 따른 추정치 차이를 비교, 분석하였다. 그리고 하위그룹별 추정치의 시계열 안정성을 분석하여 전체적인 추정치의 차이가 미미하더라도 하위그룹별 추정치는 그룹마다 차이가 있을 수 있음을 보여주었다.

본 연구는 정책과 연구의 자료로 활용되고 있는 국내 주요 보건조사의 유사점과 차이점을 제시한 연구이며 이러한 연구를 바탕으로 한 개의 조사 자료만으로 정책이나 연구의 근거로 삼아 발생할 수 있는 오류를 예방하는데 기여할 수 있을 것이다. 근래의 조사간 비교연구는 국가내 조사간 추정치 비교뿐만 아니라 국가간 조사 추정치 비교로 확대되고 있다[24]. 앞으로 국내 조사의 비교 연구와 더불어 타국가 조사와의 비교 연구를 통해 국가간 정책 비교 및 공유가 가능해질 수 있는 연구의 기틀이 마련되어야 할 것이다.

Volume: 41, Article ID: e2019012
https://doi.org/10.4178/epih.e2019012

윤리성명

본 연구는 서울대학교 생명윤리위원회의에서 심의 면제 승인을 받았습니다. (IRB No. E1711/003-002)

참고문헌

1. World Health Organization. STEPS: A framework for surveillance 2003 [cited 2018 Oct 2]. Available from: http://www.who.int/ncd_surveillance.
2. Carlson SA, Densmore D, Fulton JE, Yore MM, Kohl HW 3rd. Differences in physical activity prevalence and trends from 3 US surveillance systems: NHIS, NHANES, and BRFSS. J Phys Act Health 2009;6:S18-S27.
3. Fahimi M, Link M, Mokdad A, Schwartz DA, Levy P. Tracking chronic disease and risk behavior prevalence as survey participation declines: statistics from the behavioral risk factor surveillance system and other national surveys. Prev Chronic Dis 2008;5:A80.
4. Korea Center for Disease Control and Prevention. Community Health Survey [cited 2017 Oct 31]. Available from: https://chs.cdc.go.kr/chs/.
5. Korea Center for Disease Control and Prevention. Korea National Health and Nutrition Examination Survey [cited 2017 Oct 31]. Available from: https://knhanes.cdc.go.kr/knhanes/.
6. Nelson DE, Holtzman D, Bolen J, Stanwyck CA, Mack KA. Reliability and validity of measures from the Behavioral Risk Factor Surveillance System (BRFSS). Soz Praventivmed 2001;46 Suppl 1:S3-S42.
7. Salomon JA, Nordhagen S, Oza S, Murray CJ. Are Americans feeling less healthy? The puzzle of trends in self-rated health. Am J Epidemiol 2009;170:343-351.
8. Miller JW, Gfroerer JC, Brewer RD, Naimi TS, Mokdad A, Giles WH. Prevalence of adult binge drinking: A comparison of two national surveys. Am J Prev Med 2004;27:197-204.
9. Yun S, Zhu B, Black W, Brownson R. A comparison of national estimates of obesity prevalence from the behavioral risk factor surveillance system and the National Health and Nutrition Examination Survey. Int J Obes 2006;30:164-170.
10. Kim M, Oh G-J, Lee Y-H. Gender-Specific Factors Associated with Suicide Attempts among the Community-Dwelling General Population with Suicidal Ideation: the 2013 Korean Community Health Survey. J Korean Med Sci 2016;31:2010-2019.
11. Lee Y-H, Ko JS. Factors Associated with Smoking Cessation Intention among Current Smokers with Diabetes: Analysis of the 2013 Community Health Survey in Korea. Korean J Health Promot 2017;17:184-192.

12. Park JH, Lim SJ, Yim ES, Kim YD, Chung WJ. Factors Associated with Poor Glycemic Control among Patients with Type 2 Diabetes Mellitus: The Fifth Korea National Health and Nutrition Examination Survey (2010-2012). Health Policy and Management 2016;26:125-134.
13. Park NY, Han YH, Hyun T. Nutrient Intake of the Residents in Chungbuk Province Based on 2010 – 2013 Korea National Health and Nutrition Examination Survey. J Human Ecol 2017;21:1-13.
14. World Health Organization. International Obesity Task Force. The Asia-Pacific perspective: Redefining obesity and its treatment. WHO Western Pacific Region. 2000.
15. Li C, Balluz LS, Ford ES, Okoro CA, Zhao G, Pierannunzi C. A comparison of prevalence estimates for selected health indicators and chronic diseases or conditions from the Behavioral Risk Factor Surveillance System, the National Health Interview Survey, and the National Health and Nutrition Examination Survey, 2007-2008. Prev Med 2012;54:381-387.
16. Park YR, Cho YG, Kang JH, Park HA, Kim KW, Hur YI, et al. Comparison of Obesity and Overweight Prevalence Among Korean Adults According to Community Health Survey and Korea National Health and Nutrition Examination Survey. Korean J Obes 2014;23:64-68.
17. Yoon K, Min K, Chun H, Jang S-N, Cho S-I. Effect change of obesity on diabetes depending on measurement: self-reported body mas index from 2012 Community Health Survey vs. directly measured from the Korea National Health and Nutrition Examination Survey. Epidemiol Health 2015;37:e2015001.
18. Pierannunzi C, Hu SS, Balluz L. A systematic review of publications assessing reliability and validity of the Behavioral Risk Factor Surveillance System (BRFSS), 2004–2011. BMC Med Res Methodol 2013;13:49.
19. Kim YT, Choi BY, Lee KO, Kim H, Chun JH, Kim SY, et al. Overview of Korean Community Health Survey. J Korean Med Assoc 2012;55:74-83.
20. Korea Center for Disease Control and Prevention. Survey question guideline for Community Health Survey 2015 [cited 2017 Oct 31]. Available from: https://chs.cdc.go.kr/chs/sub03/sub03_04_01.do (Korean, authors' translation).
21. Korea Center for Disease Control and Prevention. Health Questionnaire guideline for Korea National Health and Nutrition Examination Survey 2015 [cited 2017 Oct 31]. Available from: https://knhanes.cdc.go.kr/knhanes/sub04/sub04_02_02.do?classType=4 (korean, authors' translation).
22. Beckett M, Weinstein M, Goldman N, Yu-Hsuan L. Do health interview surveys yield reliable data on chronic illness among older respondents? Am J Epidemiol 2000;151:315-323.
23. Lee S, Grant D. The effect of question order on self-rated general health status in a multilingual survey context. Am J Epidemiol 2009;169:1525-1530.

Volume: 41, Article ID: e2019012
https://doi.org/10.4178/epih.e2019012

24. Mindell JS, Moody A, Vecino-Ortiz AI, Alfaro T, Frenz P, Scholes S, et al. Comparison of Health Examination Survey Methods in Brazil, Chile, Colombia, Mexico, England, Scotland, and the United States. Am J Epidemiol 2017;186:648-658.

Volume: 41, Article ID: e2019012
https://doi.org/10.4178/epih.e2019012

Table 1. Participants aged 19 years or older in the 2010-2015 Korea Community Health Survey (KCHS) and Korea National Health and Nutrition Examination Survey (KNHANES)

Year	KCHS		KNHANES		Ratio (CHS/KNHANES)
	N	Weighted N	N	Weighted N	
2010	229126	38999317	6254	38226440	1.02
2011	229186	39904832	6027	39050481	1.02
2012	228899	40363395	5611	39674018	1.02
2013	228764	40781906	5362	40164541	1.02
2014	228695	41143286	5040	40767231	1.01
2015	228558	41554658	5571	41176539	1.01

Volume: 41, Article ID: e2019012
https://doi.org/10.4178/epih.e2019012

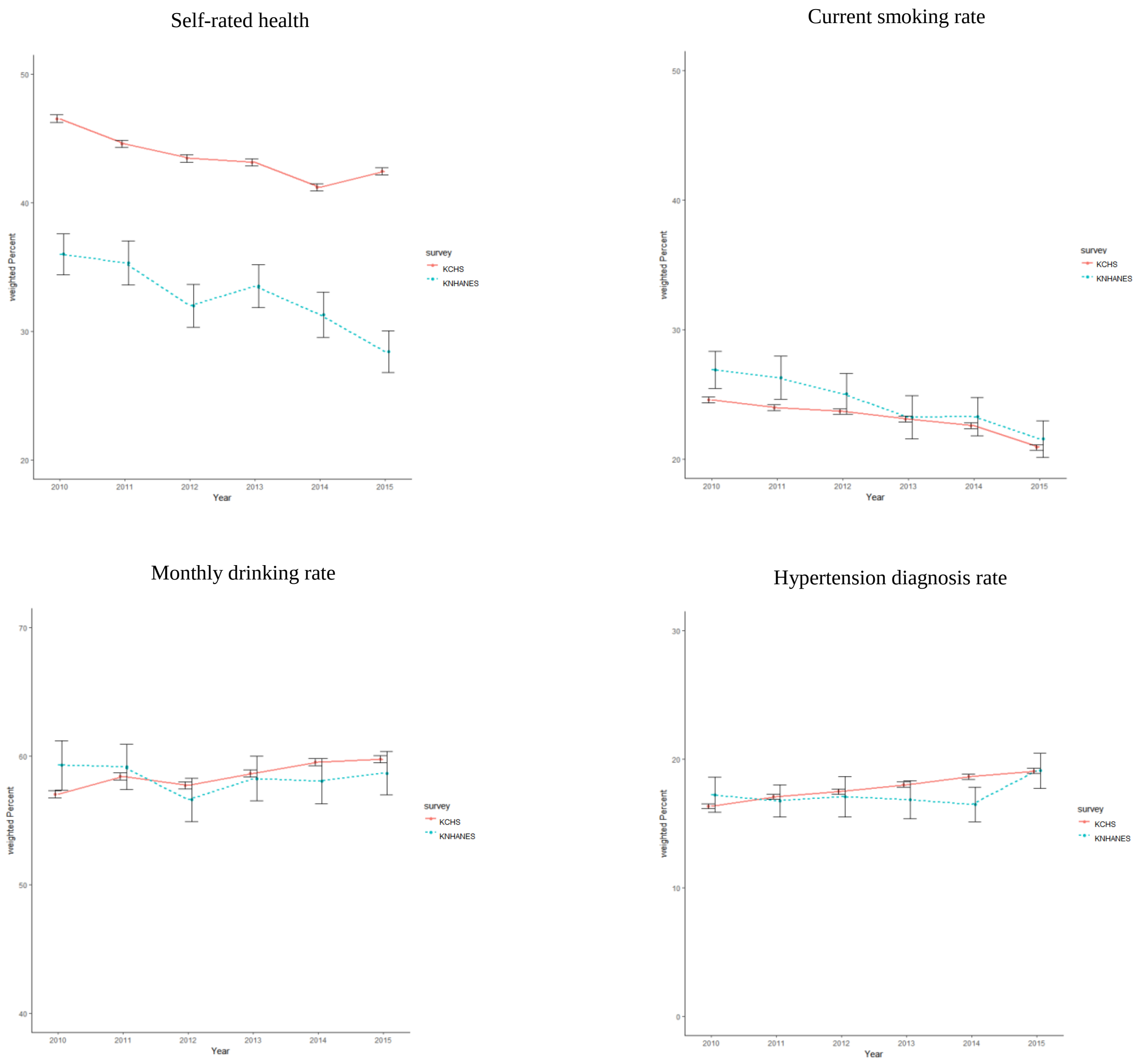

Figure 1. Trends in the variable estimates of Korea Community Health Survey (KCHS) and Korea National Health and Nutrition Examination Survey (KNHANES), 2010-2015.
(*continued to the next page*)

Diabetes diagnosis rate

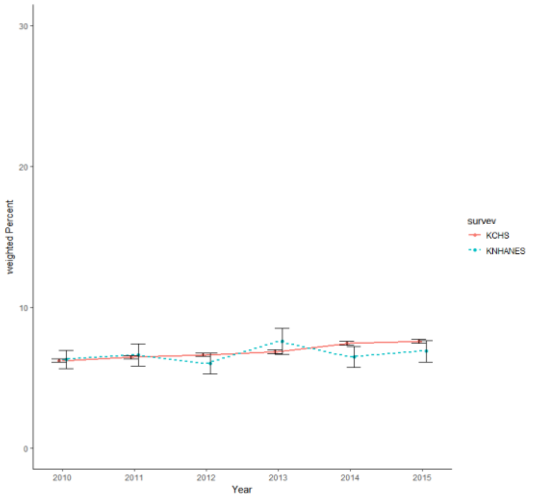

Obesity prevalence (Self-reported height & weight, KCHS) VS Obesity prevalence(Actually measured height & weight, KNHANES)

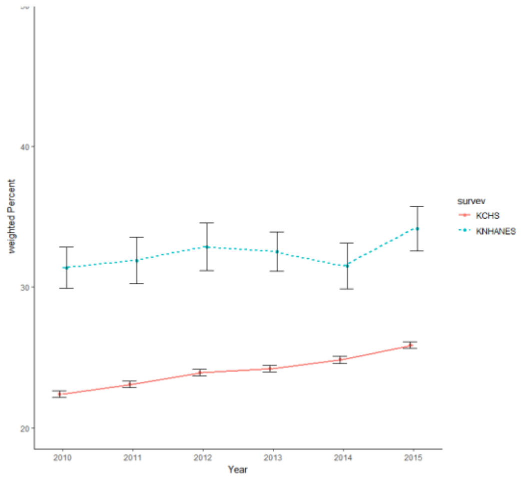

Hypertension diagnosis rate (interview, KCHS) VS Prevalence of hypertension (physical exam, KNHANES)

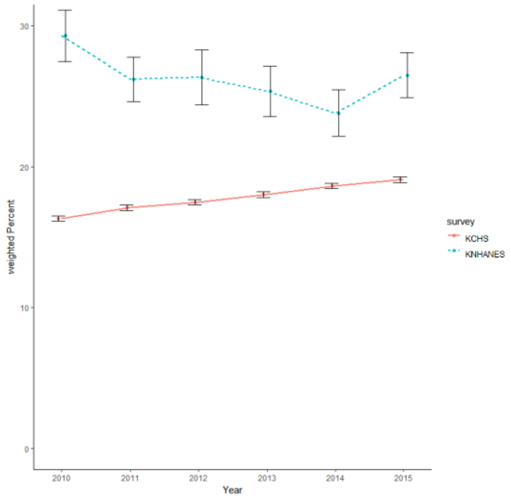

Diabetes diagnosis rate (interview, KCHS) VS Prevalence of diabetes(physical exam, KNHANES)

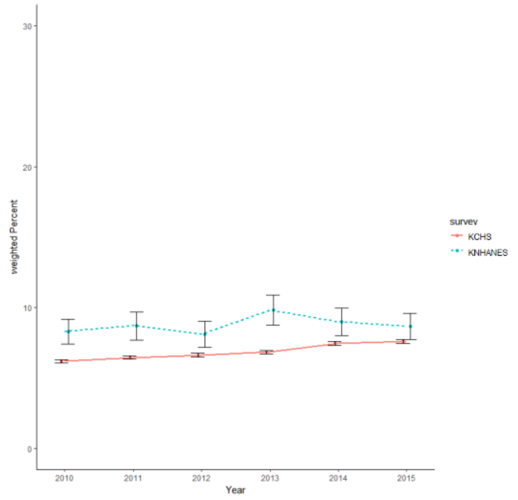

Figure 1. Continued.

Volume: 41, Article ID: e2019012
https://doi.org/10.4178/epih.e2019012

Table 2. Range of difference, mean of difference and relative difference between the estimates of Korea Community Health Survey(KCHS) and Korea National Health and Nutrition Examination Survey(KNHANES), 2010-2015

Variable	Range of Difference (%)	Mean Difference (%)	95% Confidence interval(%)	Relative difference (%)†
Self- rated health	9.2~14	10.8	9.1 to 12.5	33.0
Current smoking rate	0.1~2.3	1.2	-0.3 to 2.8	4.9
Monthly drinking rate	0.4~2.2	1.1	-0.6 to 3.0	1.9
Hypertension diagnosis rate	0.0~2.1	0.8	-0.6 to 2.2	4.6
Diabetes diagnosis rate	0.1~1.0	0.6	-0.2 to 1.3	9.0
Obesity prevalence(self-reported height & weight, KCHS) VS Obesity prevalence(Actually measured height & weight, KNHANES)	6.6~8.9	8.3	6.7 to 9.9	25.6
Hypertension diagnosis rate(interview, KCHS) VS Prevalence of hypertension(physical exam, KNHANES)	5.1~12.9	8.4	6.7 to 10.2	32.0
Diabetes diagnosis rate(interview, KCHS) VS Prevalence of diabetes(physical exam, KNHANES)	1.1~3.0	1.9	0.9 to 2.9	21.6

Values are presented as %.
Note. Mean difference(Absolute difference) is the average estimate of the differences for each year.
†Relative difference = mean difference (%, absolute difference) divided by average estimate(%) of 6-year of KNHANES

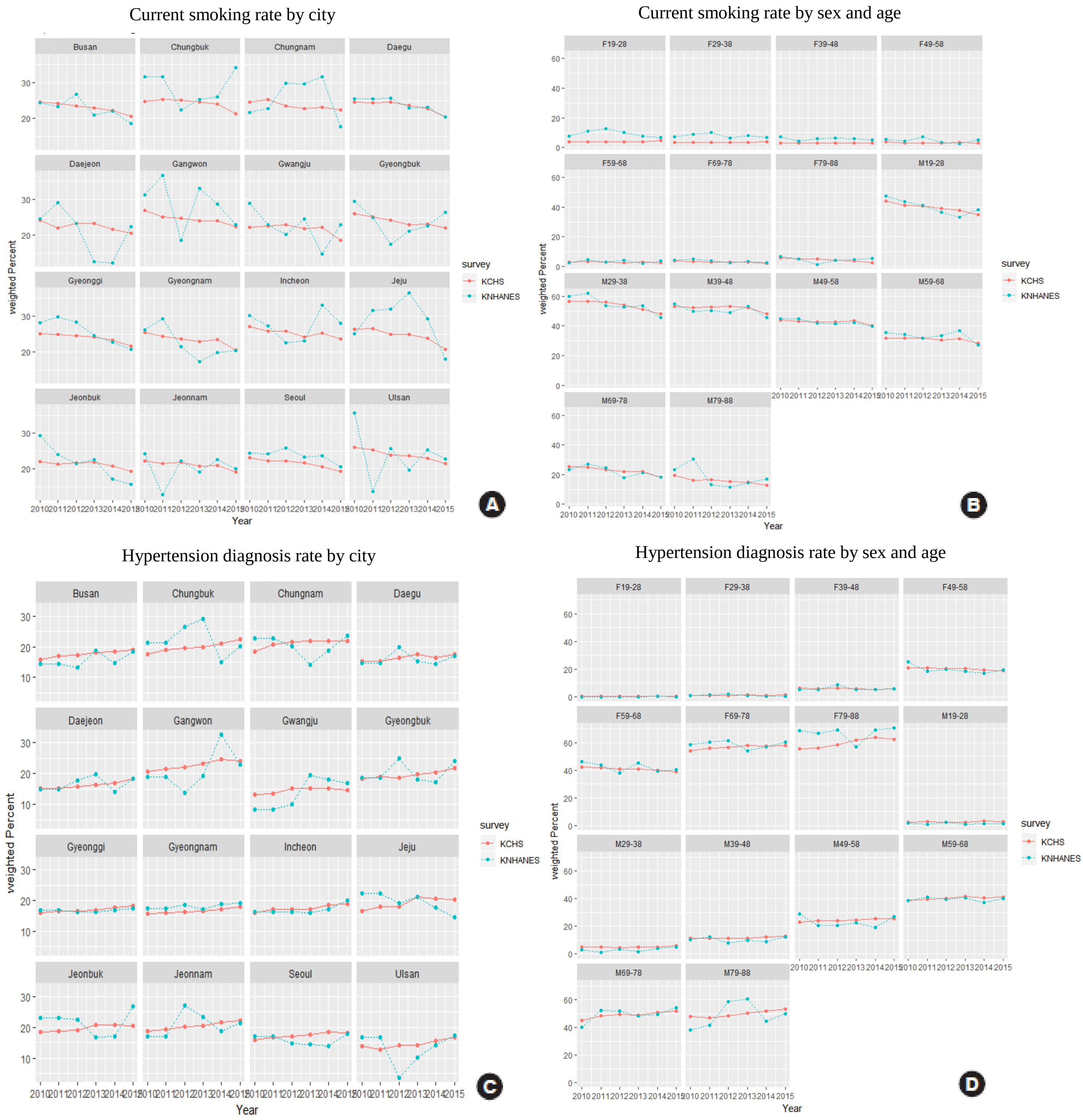

Figure 2. Time series trends (current smoking rate, hypertension diagnosis rate, and obesity prevalence) by city (A, C, and E) and by sex and age (B, D, and F) in Korea Community Health Survey (KCHS) and Korea National Health and Nutrition Examination Survey (KNHANES), 2010-2015. F, female; M, male. *(continued to the next page)*

Volume: 41, Article ID: e2019012
https://doi.org/10.4178/epih.e2019012

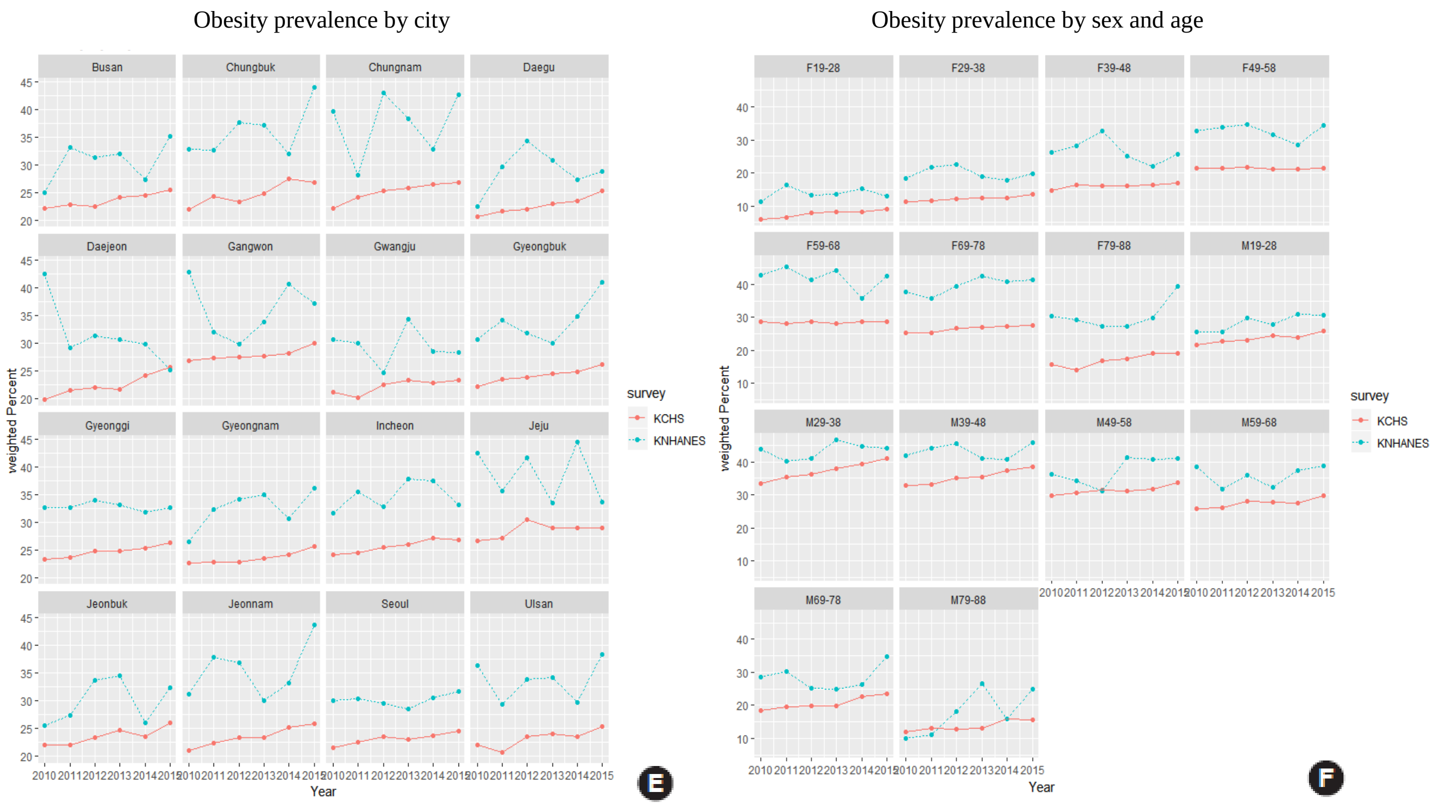

Figure 2. Continued.

Volume: 41, Article ID: e2019012
https://doi.org/10.4178/epih.e2019012

Table 3. Mean square error by city, sex and age in Korea Community Health Survey(KCHS) and Korea National Health and Nutrition Examination Survey(KNHANES), 2010-2015.

	KCHS			KNHANES		
City	Current smoking rate	Hypertension diagnosis rate	Obesity prevalence	Current smoking rate	Hypertension diagnosis rate	Obesity prevalence
Busan	0.2	0.0	0.1	4.4	4.0	13.7
Chungbuk	1.0	0.1	0.9	25.8	34.4	15.8
Chungnam	0.4	0.6	0.3	39.2	15.1	40.6
Daegu	0.7	0.3	0.1	1.0	6.3	17.6
Daejeon	0.9	0.2	0.5	38.7	6.1	16.5
Gangwon	0.2	0.2	0.3	46.7	35.5	32.2
Gwangju	1.6	0.5	0.6	18.6	8.8	12.5
Gyeongbuk	0.1	0.2	0.1	20.3	13.4	10.6
Gyeonggi	0.2	0.1	0.1	2.4	0.2	0.6
Gyeongnam	0.6	0.1	0.3	11.3	1.9	8.5
Incheon	0.4	0.1	0.1	20.3	2.1	7.2
Jeju	1.0	1.0	1.6	46.7	3.7	27.1
Jeonbuk	0.4	0.2	0.5	2.8	19.5	17.0
Jeonnam	0.2	0.0	0.1	20.0	21.4	25.3
Seoul	0.1	0.1	0.1	1.8	3.1	1.2
Ulsan	0.1	0.5	0.8	61.1	33.0	15.6
Mean	0.5	0.3	0.4	22.6	13.0	16.4

Volume: 41, Article ID: e2019012
https://doi.org/10.4178/epih.e2019012

Table 3. continued.

Group(sex age)	KCHS			KNHANES		
	Current smoking rate	Hypertension diagnosis rate	Obesity prevalence	Current smoking rate	Hypertension diagnosis rate	Obesity prevalence
F 19-28	0.0	0.0	0.1	5.9	0.0	3.8
F 29-38	0.0	0.0	0.1	2.1	0.3	4.4
F 39-48	0.0	0.1	0.2	1.0	2.4	13.5
F 49-58	0.1	0.1	0.1	3.1	5.8	5.8
F 59-68	0.0	0.1	0.1	1.1	9.7	11.1
F 69-78	0.0	0.5	0.1	0.4	8.7	3.1
F 79-88	0.1	2.1	1.2	3.7	30.8	17.9
M 19-28	0.3	0.3	0.3	9.4	9.4	1.8
M 29-38	1.8	0.1	0.1	8.1	1.8	5.8
M 39-48	2.0	0.2	0.3	8.8	4.4	6.6
M 49-58	0.9	0.1	0.3	0.9	19.6	12.1
M 59-68	0.9	0.5	0.6	10.7	2.5	10.8
M 69-78	0.8	1.1	0.7	7.5	18.9	16.1
M 79-88	0.8	1.0	0.8	41.7	89.7	25.6
Mean	0.6	0.4	0.3	7.0	13.6	9.2
